# Knockdown of MALAT1 expression inhibits HUVEC proliferation by upregulation of miR-320a and downregulation of FOXM1 expression

**DOI:** 10.18632/oncotarget.18507

**Published:** 2017-06-16

**Authors:** Jian-Yong Sun, Zheng-Wei Zhao, Wei-Miao Li, Guang Yang, Peng-Yu Jing, Pei Li, Hai-Zhou Dang, Zhao Chen, Yong-An Zhou, Xiao-Fei Li

**Affiliations:** ^1^ Department of Thoracic Surgery, Tangdu Hospital, Fourth Military Medical University, Xi’an, Shaanxi, China; ^2^ Department of Respiration, Tangdu Hospital, Fourth Military Medical University, Xi’an, Shaanxi, China

**Keywords:** MALAT1, HUVECs, proliferation, miR-320a, FOXM1

## Abstract

Regulation of cancer angiogenesis could be a useful strategy in cancer therapy. Metastasis-associated lung adenocarcinoma transcript 1 (MALAT1) is a long non-coding RNA (lncRNA), and can induce cancer cell proliferation, while lncRNAs, generally are able to act as microRNA (miRNA) sponges. The latter is a type of competitive endogenous RNA (ceRNA) that regulates expression of the targeting miRNAs and protein-coding genes. This study investigated the proliferative role of MALAT1 in human umbilical vein endothelial cells (HUVECs) and the underlying molecular events. The data showed that knockdown of MALAT1 expression using MALAT1 siRNA inhibited HUVEC proliferation and also significantly decreased levels of FOXM1 mRNA and protein *in vitro*, while knockdown of FOXM1 expression reduced HUVEC proliferation. Annotation of HUVEC microarray data revealed that seven miRNAs, including miR-320a, were upregulated after knockdown of MALAT1 expression in HUVECs. MALAT1 was shown to reciprocally interact with miR-320a, i.e., expression of one negatively regulated levels of the other, whereas knockdown of MALAT1 expression promoted miR-320a levels. Furthermore, miR-320a could directly target and inhibit FOXM1 expression in HUVECs. Knockdown of MALAT1 expression enhanced miR-320a expression but reduced FOXM1 expression resulting in downregulation of HUVEC proliferation. However, such an effect was inhibited by miR-320a depletion. In conclusion, this study demonstrates that miR-320a plays an important role in mediating the effects of MALAT1 on HUVEC proliferation by suppression of FOXM1 expression. Thus, targeting of this gene pathway could be a novel strategy in cancer therapy.

## INTRODUCTION

Angiogenesis is an essential physiological process in formation of new blood vessels in the human body and plays an important role in tissue growth and development as well as wound healing and tumorigenesis [1]. Blood vessels not only transport oxygen and nutrients throughout the human body but also nourish tumors and other disease conditions [2]. To date, the precise mechanisms of angiogenesis remain to be defined but a large number of studies have shown that mechanical and chemical stimulation could regulate expression of different cytokine and growth factors to promote endothelial cell proliferation, while angiogenesis is also the key process in cancer development and progression [3]. Malignant cells are rapidly dividing cells and grow uncontrollably due to activation of oncogenes and/or inactivation of tumor suppressor genes [4]. During and after tumors have formed to a size of 1-2 mm^3^, the blood supply cannot provide the oxygen and nutrients in order to further facilitate their growth [5]. Tumor cells can, therefore, promote angiogenesis through expression and secretion of various growth factors, such as basic fibroblast growth factor (bFGF) or vascular endothelial growth factor (VEGF) [6]. Angiogenesis could thus serve as a therapeutic target in the control of human cancers through anti-angiogenesis and in control of cardiovascular diseases by promotion of angiogenesis [7]. Further investigation could provide a better understanding of regulatory molecules in pathological angiogenesis and could aid in the discovery of a potential anti-cancer treatment. Currently, there are several agents or antibodies used in clinical trials for treatment of various human cancers, such as lung, breast, and colorectal cancers [8, 9].

Indeed, angiogenesis is shown to be regulated by different genes [10] and published data have revealed that non-coding RNAs (ncRNA), including microRNAs (miRNAs) and long non-coding RNAs (lncRNAs), can regulate gene expression post-transcriptionally. Furthermore, they could also regulate expression of angiogenesis-related genes [11]. lncRNAs are non-coding RNA transcripts that exceed 200 nucleotides in length and accumulating evidence shows that altered lncRNA expression contributes to human tumorigenesis [12], e.g., metastasis-associated lung adenocarcinoma transcript 1 (MALAT1), a large, infrequently spliced non-coding RNA, is aberrantly expressed in various cancers [13]. Moreover, miRNAs are small, non-protein coding RNA transcripts approximately 18–25 nucleotides in length that also regulate mRNA translation in cells [14] and aberrant miRNA expression has been robustly associated with human cancer development and progression [15]. Study of the relationship of these two types of ncRNAs demonstrated that the lncRNA CRNDE, acted as a competitive endogenous RNA (ceRNA) of miR-384, resulting in promotion of proliferation, migration, and invasion of hepatic carcinoma cells [16].

Furthermore, Forkhead box protein M1 (FOXM1), also known as HFH-11(HNF-3/fork head homolog 11), MPP-2(methoxyphenyl piperazine-2), belongs to a family of transcriptional factors that regulate a wide spectrum of metabolic and developmental functions [17]. FOXM1 has a diverse effect on eukaryotic cells, including regulation of many cell cycle G2/M phase-specific genes [18]. Furthermore, FOXM1 downregulation leads to pleiotropic cell cycle defects, such as a delay in G2, abnormal chromosome segregation and frequent failure of cytokinesis [19]. Downregulation of FOXM1 also enabled *Propionibacterium* acnes (*P. acnes*) to exert a profound effect on cell cycle progression, most likely due to perturbations of kinetochore and centromere assembly and functionality [20]. Potential significance of FOXM1 in angiogenesis and HUVEC proliferation was also established in previous studies [21, 22], although the underlying molecular pathway remains elusive.

In this study, we investigated the proliferative role of MALAT1 in HUVECs and the underlying molecular events. The rationale for us to link and explore MALAT1, miR-320a, and FOXM1 in HUVECs was because our previous study of miRNA profiling in HUVECs after knockdown of MALAT1 expression demonstrated that up-regulation of miR-320a could target FOXM1. This study is expected to provide a novel target for future anti-angiogenesis therapy of human cancer.

## RESULTS

### Inhibition of HUVEC proliferation after knockdown of MALAT1 expression *in vitro*

**Figure 1:**
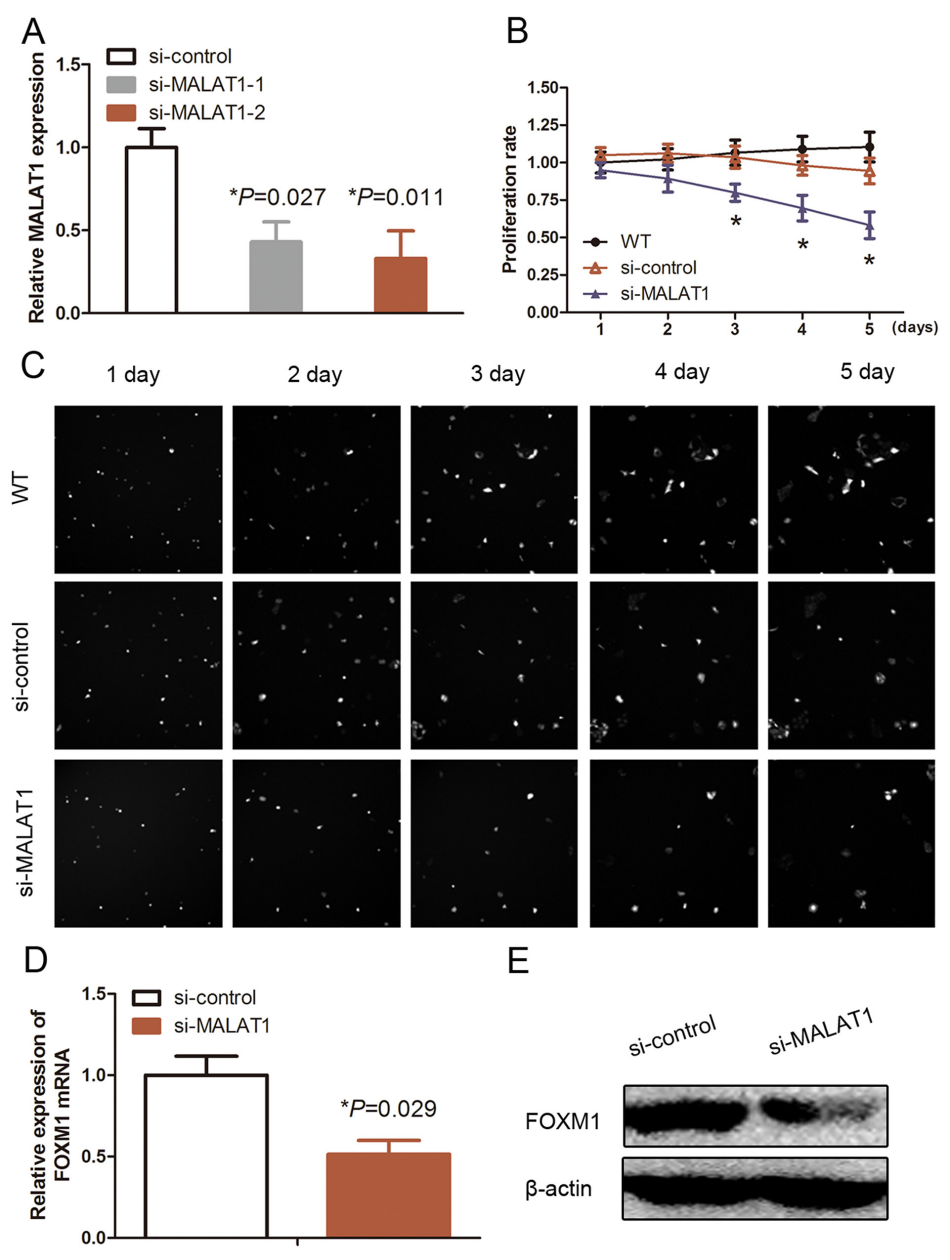
Effects of MALAT1 siRNA on inhibition of MALAT1 expression and cell proliferation **(A)** qRT-PCR. HUVECs were grown and transfected with MALAT1 siRNA (#1 and #2) or scrambled siRNA and then subjected to qRT-PCR analysis of MALAT1 expression (n=3). **(B)** and **(C)** Cell proliferation assay. HUVECs were grown and transfected with MALAT1 siRNA or scrambled siRNA and then subjected to the cell proliferation assay. The graph in B is quantified data from C. **(D)** qRT-PCR. HUVECs were grown and transfected with MALAT1 siRNA (#1) or scrambled siRNA and then subjected to qRT-PCR analysis of FOXM1 expression (n=3). **(E)** Western blot. HUVECs were grown and transfected with MALAT1 siRNA or scrambled siRNA and then subjected to immunoblotting analysis of FOXM1 expression.

MALAT1 was reported to promote cell proliferation, thus, we further explored the effect of MALAT1 on HUVECs by knockdown of MALAT1 expression using MALAT1 siRNAs. As shown in Figure 1A, siMALAT1 (#2) had a high efficiency for knockdown of MALAT1 expression in HUVECs compared with si-MALAT1 (#1). Thus, si-MALAT1 (#2) was used for all of our experiments in this study (Figure 1A; *P*<0.05). The data on the *in vitro* real-time cell proliferation assay showed a significant decrease in viability of si-MALAT1-transfected HUVECs compared with si-control–transfected cells (Figure 1B and 1C; *P*<0.05). Based on previous studies, we identified that FOXM1 is the key cell proliferation regulation molecule during HUVEC proliferation. We thus performed qRT-PCR and Western blotting to detect changed levels of FOXM1 in si-MALAT1-transfected HUVECs and found a significant decrease in FOXM1 mRNA and protein after knockdown of MALAT1 expression in HUVECs (Figure 1D and 1E).

### Inhibition of HUVEC proliferation after knockdown of FOXM1 expression *in vitro*

**Figure 2:**
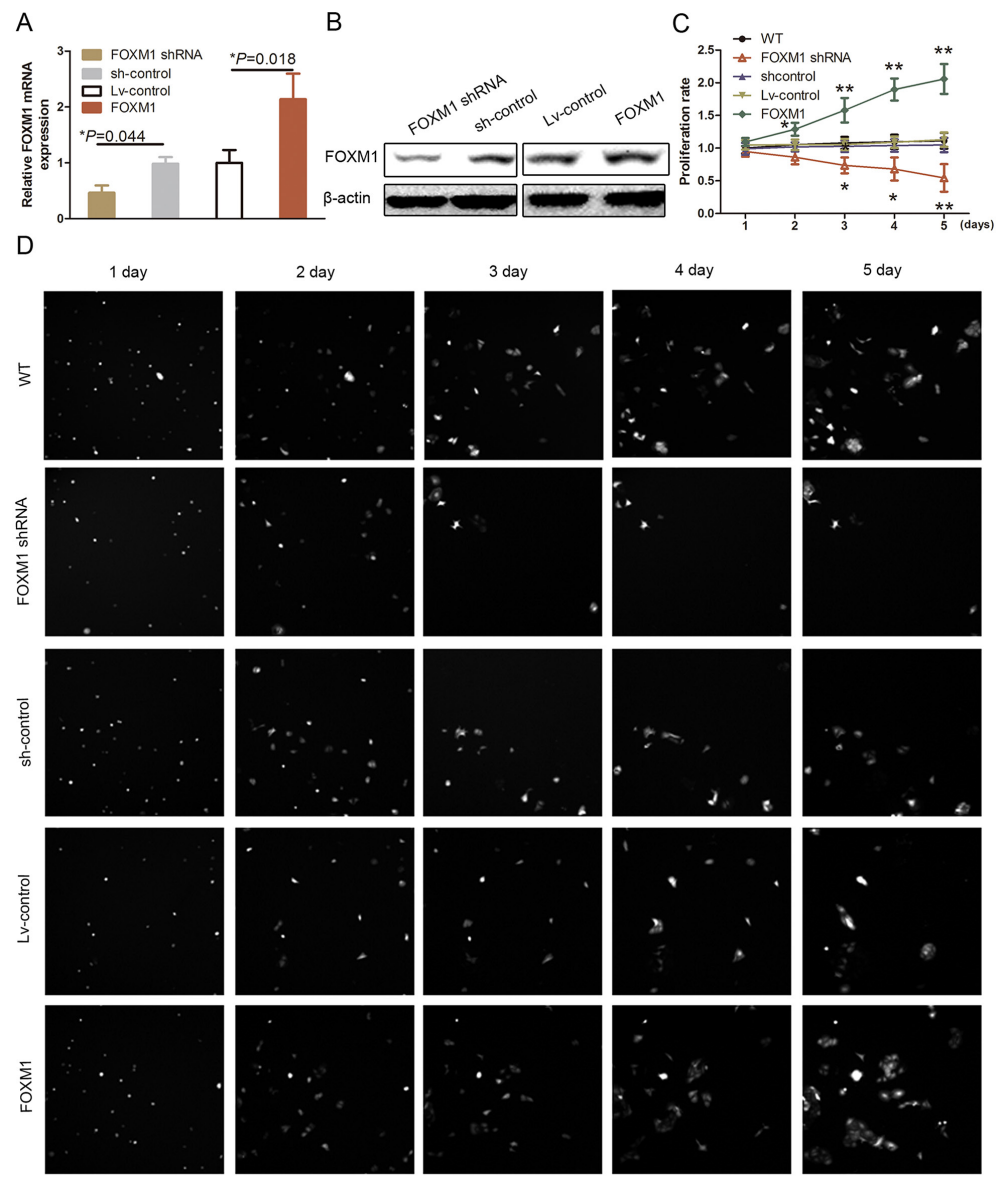
Effect of FOXM1 knockdown on regulation of HUVEC proliferation **(A)** qRT-PCR. HUVECs were grown and transfected with FOXM1 or Lv-control (lentivirus-control), shRNA or scrambled shRNA and then subjected to qRT-PCR analysis of FOXM1 expression. **(B)** Western blot. HUVECs were grown and transfected with FOXM1 shRNA or scrambled siRNA and then subjected to immunoblotting analysis of FOXM1 expression. **(C)** and **(D)** Cell proliferation assay. HUVECs were grown and transfected with FOXM1 shRNA or scrambled shRNA and then subjected to the cell proliferation assay. The graph in C is quantified data from D.

To confirm whether MALAT1 siRNA-reduced HUVEC proliferation was associated with FOXM1 downregulation in HUVECs and explore the biological functions of FOXM1, we transfect FOXM1 cDNA, shRNA, or control into HUVECs (Figure 2A and 2B; *P*<0.05). Our data showed that knockdown of endogenous FOXM1 expression using FOXM1 shRNA significantly reduced HUVEC viability (Figure 2C and 2D; *P*<0.05).

### Reciprocal interaction between MALAT1 and miR-320a in HUVECs

**Figure 3:**
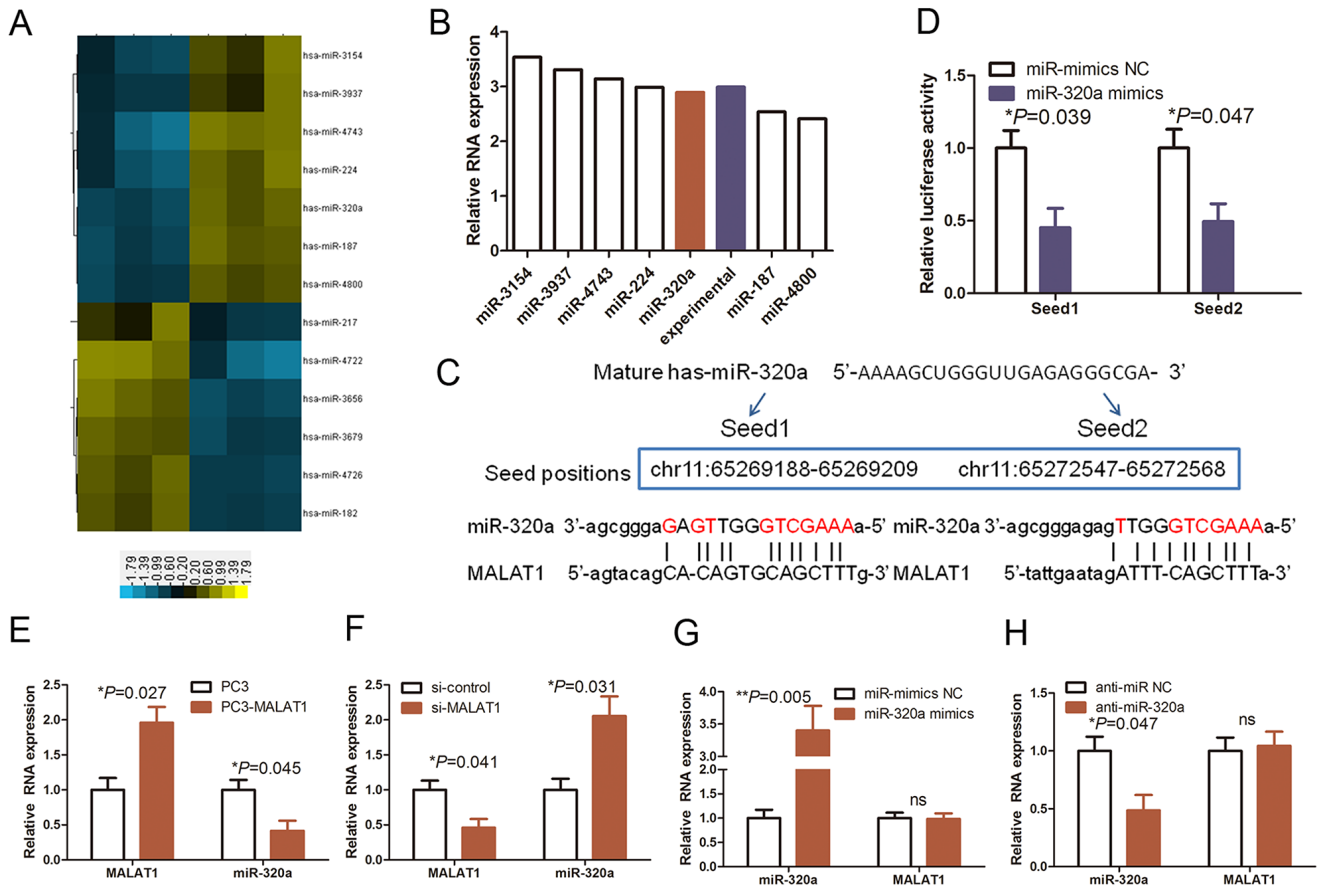
The reciprocal interaction of MALAT1 and miR-320a in HUVECs **(A)** The data on miRNA profiling in MALAT1 knocked down HUVECs. Our previous study assessed differentially expressed miRNAs between MALAT1 siRNA and control siRNA-transfected HUVECs using miRNA array. The shaded yellow section represents increased miRNA expression, whereas the shaded blue section represents decreased miRNA expression. **(B)** The graph shows the top seven upregulated miRNAs and were confirmed by qRT-PCR. **(C)** Prediction and alignment of the potential miR-320a binding regions to MALAT1. **(D)** Luciferase reporter assay. Relative luciferase activity mediated by the reporter constructs harboring the miR-320 binding site upon transfection with miR-320a mimics or NC. **(E)** and **(F)** qRT-PCR. HUVECs were grown and transfected with MALAT1 cDNA (PC3-MALAT1) or vector-only plasmid or MALAT1 siRNA (si-MALAT1) or negative control siRNA and then subjected to qRT-PCR analysis of MALAT1 and miR-320a levels. **(G)** and **(H)** qRT-PCR. HUVECs were grown and transfected with miR-320 mimics, anti- miR-320 or their NC controls and then subjected to qRT-PCR analysis of MALAT1 and miR-320a levels.

A previous study reported that lncRNAs could function as ceRNA or “molecular sponges” to modulate miRNA expression and functions [13]. During human carcinogenesis, there are a large number of miRNAs, which are aberrantly expressed and functionally identified as pro- and anti-proliferation miRNAs [16, 23]. To explore how MALAT1 knockdown reduced HUVEC proliferation that was linked to the modulation of miRNA expression, we analyzed the miRNA profiling data in MALAT1 knockdown HUVECs (Figure 3A) and found seven miRNAs that were upregulated in MALAT1 knockdown HUVECs (Figure 3B), including miR-320a. Thus, we selected miR-320a for further study, which was based on 1) it is a well-known cell-cycle miRNA, expression of which was shown to be frequently downregulated in multiple cancers and our laboratory has previously reported that miR-320 functions as proliferation suppressor gene in colon cancer [23] and 2) miR-320a could bind to MALAT1 according to RNAhybrid 2.234 (https://bibiserv.cebitec.uni-bielefeld.de/rnahybrid/) analysis.

We initially assayed the direct binding of miR-320 to the MALAT1 promoter by construction of luciferase vectors carrying potential miR-320 binding sites (Seed 1 and 2; Figure 3C and 3D; *P*<0.05). As shown in Figure 3C, the luciferase activity was significantly decreased in miR-320a-transfected cells compared with that of the control vector-transfected cells. Indeed, MALAT1 cDNA transfection significantly increased MALAT1 expression but reduced miR-320a expression in HUVECs (Figure 3E; *P*<0.05), whereas knockdown of MALAT1 expression increased miR-320a expression (Figure 3F; *P*<0.05). miR-320a overexpression or knockdown did not change MALAT1 levels in HUVECs (Figure 3G and 3H). These results suggest that interaction of MALAT1 with miR-320a has reciprocal effects.

### miR-320a directly targets and regulates FOXM1 expression in HUVECs

**Figure 4:**
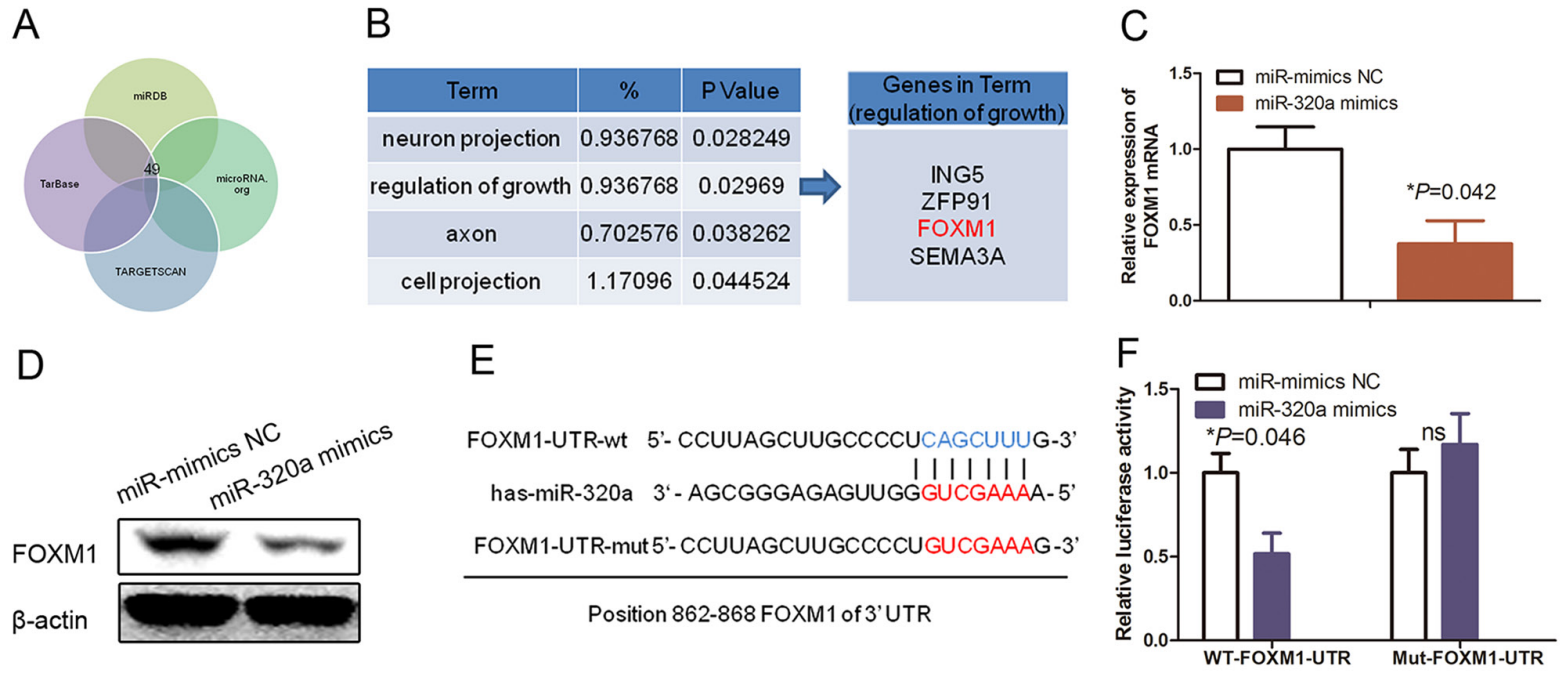
FOXM1 as a direct target of miR-320a in HUVECs **(A)** Gene Ontology classification of the predicted miR-320a target genes after integration of four algorithms (Targetscan, TarBase, miRDB, and microRNA.org). **(B)** The GO classification of miR-320a targets predicted by integration of the four algorithms. **(C)** qRT-PCR. HUVECs were grown and transfected with miR-320a mimics or NC and then subjected to qRT-PCR analysis of FOXM1 expression. **(D)** Western blot. HUVECs were grown and transfected with miR-320a mimics or NC and then subjected to Western blot analysis of FOXM1 expression. **(E)** Prediction of the miR-320a binding sites to the FOXM1 3’-UTR using the sequence complementarity and phylogenic conservation of 7/8-nucleotide seed sequence prediction. **(F)** Luciferase reporter assay. The relative luciferase activity mediated by the reporter constructs harboring the wild type (wt) or mutated FOXM1 3’-UTR upon transfection with miR-mimics NC or miR-320a mimics.

To explore the underlying molecular mechanism by which miR-320a regulated HUVEC proliferation, we searched for potential targets of miR-320a in the human genome database using different bioinformatic miRNA target prediction tools (Figure 4A). We revealed a list of candidate-targeting mRNAs from the Targetscan, TarBase, miRDB, and microRNA.org prediction tools. We then performed Gene Ontology (GO) analysis using DAVID Bioinformatics Resources (http://david.abcc.ncifcrf.gov/) to assess such candidate genes and found that these genes were functionally enriched in several biological processes, such as cell growth regulation (Figure 4B). Interestingly, FOXM1 is one of the targeting genes (Figure 4B). Thus, we performed qRT-PCR and Western blotting analysis to confirm that FOXM1 was indeed the direct target of miR-320a (Figure 4C; *P*=0.042).

Next, we constructed two dual luciferase reporters to verify the putative binding sites of miR-320a to FOXM1 (Figure 4D). In both HEK293T and HUVECs, miR-320a could effectively reduce the relative luciferase activity of the wild-type reporter, but not the reporter carrying the mutant sequence of the potential binding sites (Figure 4E and 4F; *P*<0.05). Thus, our data confirmed that FOXM1 is a functional target of miR-320a.

### Effects of miR-320a on regulation of MALAT1-knockdown suppression of HUVECs proliferation

**Figure 5:**
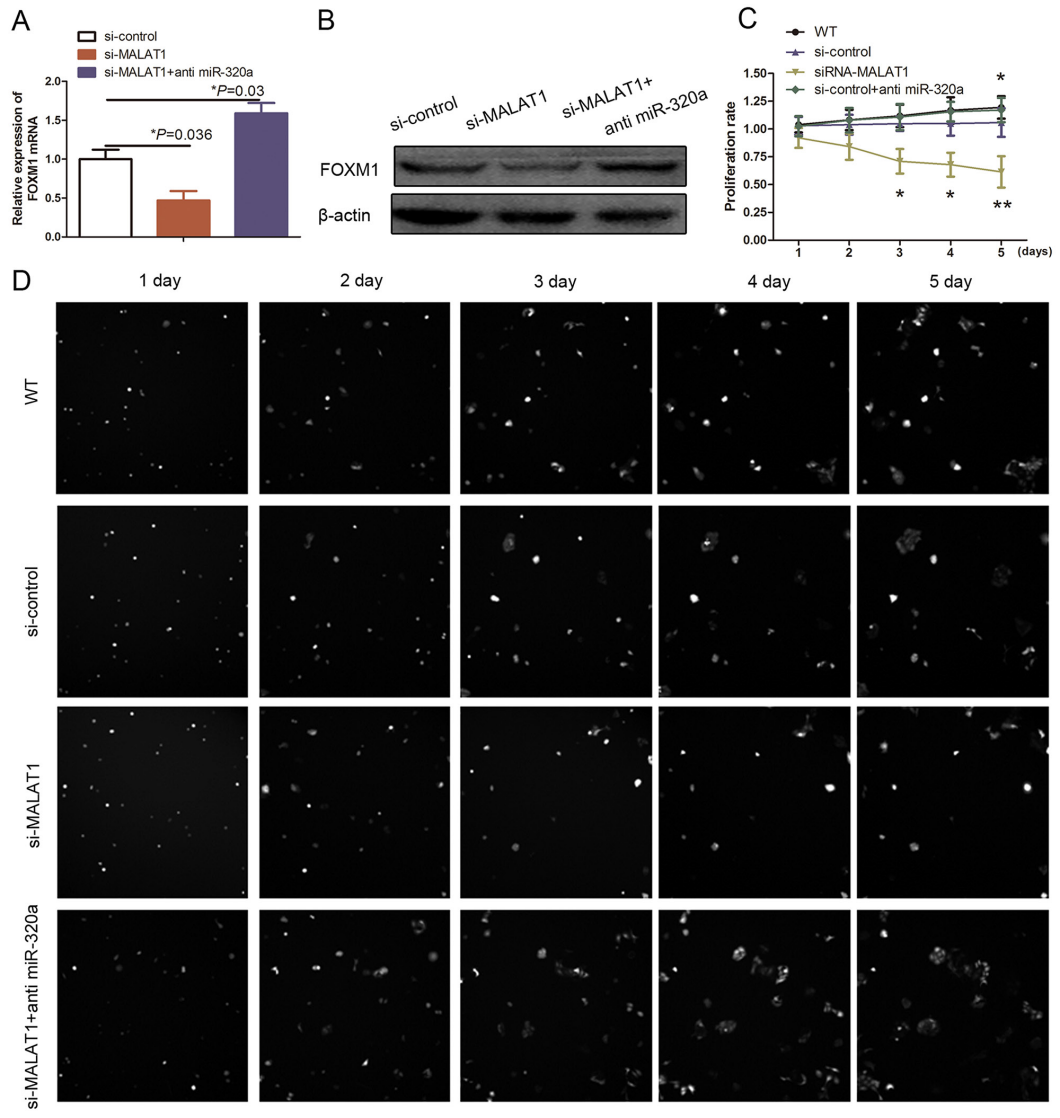
Effect of miR-320a on HUVEC proliferation **(A)** qRT-PCR. HUVECs were grown and transfected with MALAT1 siRNA, miR-320a inhibitors, or their combination and then subjected to qRT-PCR analysis of FOXM1 expression. **(B)** Western blot. HUVECs were grown and transfected with MALAT1 siRNA, miR-320a inhibitors, or their combination and then subjected to Western blot analysis of FOXM1 expression. **(C)** and **(D)** Cell proliferation assay. HUVECs were grown and transfected with MALAT1 siRNA, miR-320a inhibitors or their combination and then subjected to cell proliferation assay.

We further assessed the role of miR-320a expression in regulation of gene expression and HUVEC proliferation and found that transfection of a miR-320a inhibitor suppressed the effects of MALAT1 on inhibition of FOXM1 mRNA and protein levels (Figure 5A and 5B; *P*<0.05). Furthermore, miR-320a knockdown also significantly reversed MALAT1 knockdown-induced suppression of HUVEC viability (Figure 5C and 5D; *P*<0.05). Taken together, our current data indicate that miR-320a plays an important role in mediating the effects of MALAT1 on HUVEC growth by suppression of FOXM1 expression.

## DISCUSSION

Different blood vessel growth factors and cytokines, such as VEGF, bFGF, and transforming growth factor-β (TGF-β), regulate angiogenesis and cancer angiogenesis. Targeting of their expression, secretion, or functions have been researched as novel strategies for anti-angiogenesis therapy for different human cancers [24, 25]. However, to date, the clinical trial data are elusive [26] e.g., Avastin is an anti-VEGF inhibitor, and was approved by the US FDA in 2004 as an anti-angiogenesis drug to control breast cancer treatment. However, it was revoked on November 18, 2011 due to a lack of efficacy and unacceptably high toxicity [27]. Thus, further research and novel approaches are needed to control cancer-related angiogenesis. Our current study investigated lncRNA, miRNA, and protein-coding genes and found that MALAT1, miR-320a, and FOXM1 could form a gene signaling pathway to regulate HUVEC proliferation, which may provide a novel strategy for future control of cancer progression.

Our current study was initially promoted by and based on microarray data. Advances in the tiling array and sequencing technologies have truly helped basic and translational researchers to reveal that the majority (∼97%) of human genome sequences are transcribed into ncRNAs but not the protein-coding genes [28]. These ncRNAs can be divided into lncRNAs (-200 nt) and miRNAs (18-25 nt) according to their length of nucleic acid. In the current study, we found that knockdown of MALAT1 expression significantly downregulated the HUVEC proliferation rate, which was mediated by upregulation of miR-320a and consequent downregulation of FOMX1 expression in HUVECs. The hypothesis of our current study was based on our previous microarray data on MALAT1-knockdown HUVECs, which showed that the downregulation of MALAT1 upregulated miR-320a expression. Indeed, our current data revealed that upregulated miR-320a expression resulted in suppression of targeted FOXM1 expression and consequent inhibition of HUVEC proliferation. These data demonstrated that MALAT1 played an important role in endothelial cell proliferation and pathogenesis of cancer angiogenesis.

Although lncRNA possesses vital biological functions in cancer development and progression, the underlying molecular mechanisms underpinning the lncRNA interaction with gene expression are complex and diverse, incompletely understood [29]. Thus, much effort has focused on functional analysis of lncRNAs that are processed into short fragments, such as miRNAs, which can repress the expression of targeting protein coding genes via homologous base pairing [30]. For example, MALAT1 expression is elevated in various types of human cancer, whereas knockdown of MALAT1 expression arrests the cell cycle at the G1/S phase [31]. Thus, it has been recently speculated that lncRNAs might epigenetically regulate gene expression by competing for shared miRNA response elements, thereby acting as a natural miRNA sponge that reduces the binding of endogenous miRNAs to their target genes [32]. A previous study showed that MALAT1 was able to promote lung adenocarcinoma cell epithelial–mesenchymal transition (EMT) and metastasis by downregulation of expression of the targeting gene, SLUG, as a ceRNA for miR-204 [7]. Another study reported that MALAT1 upregulated FBXW7 expression by modulation of the MALAT1-miR-155 pathway, which might serve as a novel prognostic biomarker and therapeutic target for glioma [15]. Our current study demonstrated that MALAT1 could also target miR-320a in HUVECs.

miRNAs can inhibit expression of their targeting protein-coding genes and lncRNAs [13]. Expression of some miRNAs could act as oncogenes, whereas lost expression of other miRNAs could be tumor suppressors in cancer [33]. In this context, miR-320a is generally considered as a tumor suppressor in various human cancers, including gastric [34], breast [35], colorectal [36] and colon cancers [23]. However, it remains unknown how MALAT1 suppresses miR-320a, in addition to other miRNAs discussed in the previous sections. We thus explored the potential of MALAT1 exerting its function on miR-320a by performing microarray and bioinformatic analyses for miRNAs (including miR-320a) that had complementary-base pairing with MALAT1. We found that miR-320a was a highly likely target of MALAT1 since it was shown to be negatively regulated by MALAT1. Indeed, our previous study demonstrated that miR-320a was a growth-suppressive miRNA [23]. Thus, our current study linked MALAT1 to miR-320a in HUVECs.

Next, we performed bioinformatical analyses to reveal the miR-320a-targeting gene and found that FOXM1 is one of these. FOXM1, as a transcriptional activator, is involved in cell proliferation. FOXM1 protein is phosphorylated in the M phase of the cell cycle and regulates expression of several cell cycle genes, such as cyclinB1 and cyclinD1 [18, 19]. Inhibition of FOXM1 expression in human cancer cells could potentially suppress tumorigenesis [20]. In angiogenesis research, endothelial FOXM1 has garnered attention as a guardian of vascular angiogenesis and proliferation [21]. However, a given miRNA usually targets multiple genes and thus, it remains to be further studied whether miR-320a can target other angiogenesis-related genes, such as VEGF, bFGF, or TGF-β.

In summary, the data presented in the current study revealed a novel gene pathway. MALAT1-induced miR-320a expression suppressed FOXM1 expression and consequently inhibited HUVECs proliferation. Given the oncogenic role of MALAT1 in human cancers, MALAT1 could mediate HUVECs proliferation in human carcinogenesis and cancer progression; thus, targeting of the MALAT1-miR-320a-FOXM1 signaling could serve as a novel strategy for therapeutic intervention in HUVECs-driven angiogenesis.

## MATERIALS AND METHODS

### Cell line and culture

HUVECs were obtained from the American Type Culture Collection (ATCC, Manassas, VA, USA) and grown in RPMI-1640 medium (Gibco-BRL, Gaithersburg, MD, USA) supplemented with 10% fetal bovine serum (HyClone, Logan, UT, USA) in a humidified incubator containing 5% CO_2_ at 37°C. HUVECs were cultured and used for our experiments within 6 months.

### siRNAs and transfection

MALAT1 siRNAs were specifically designed and synthesized by Shanghai GenePharma Co, Ltd. (Shanghai, China) by targeting MALAT1 sequences (MALAT1-1, 5’-CACAGGGAAAGCGAGTGGTTGGTAA-3’ and 5’-TTACCAACCACTCGCTTTCCCTGTG-3’; and MALAT1-2, 5’-GAGGUGUAAAGGGAUUUAUTT-3’ and 5’-AUAAAUCCCUUUACACCUCTT-3’). A negative control siRNA was also synthesized with targeting sequences of 5’-GGCCUAAAGUAGUAGCUAUTT-3’ and 5’-AUAGCUACUACUUUAGGCCTT-3’.

To assess the effects of miR-320a on regulation of HUVEC proliferation, we had a miR-320a mimic, a miR-320a inhibitor, and control RNA chemically synthesized and purified using high-performance liquid chromatography (GenePharma). The concentration of these synthesized siRNAs (mimic, inhibitor, and negative control) were all 20 μM and the working concentration was 20 nM diluted at 1:1000. Plasmids containing MALAT1 siRNAs were transfected into cells for 48 h using Lipofectamine 2000 (Invitrogen, Carlsbad, CA, USA) according to the manufacturer’s instructions and then the cells were used for our experiments.

### Expression and package of FOXM1 cDNA-containing lentivirus

Plasmids-carrying human FOXM1 cDNA and shRNA were purchased from Open Biosystems (Cat#: RH54533-NM_001005366, Huntsville, AL, USA) and then used to transiently transfect into human embryonic kidney 293T (HEK293T) cells for production of lentivirus using Lipofectamine 2000 (Invitrogen) and 48 h later, the media from the HEK293T cell cultures were centrifuged at 3000 rpm for 20 min to purify lentivirus, which was then further evaluated for multiplicity of infection (MOI). To express or knockdown FOXM1 protein, HUVECs were pre-treated with DEAE dextran (25 μg/ml) for 45 min and then infected with lentiviruses at 10 MOI for 48 h. After infection, cells were selected with growth medium containing 2 μg/ml puromycin or 100 μg/ml bleomycin (Sigma-Aldrich, St. Louis, MO, USA), respectively.

### Quantitative reverse transcription-polymerase chain reaction (qRT-PCR)

Total RNA was isolated from cells using Trizol (Invitrogen) according to the manufacturer’s instructions. After quantification of the RNA samples, cDNA was synthesized from 1.0 μg of total RNA using oligo-dT primers and the retroscript reverse transcription kit (Cat#: AM1710, Ambion, Austin, TX, USA). qPCR was then performed in triplicate using the RT2 Real-Time SYBR Green PCR master mix system (Cat#: PA-110, SuperArray Bioscience Corporation, Hilden, Germany) in an Opticon2 DNA Monitor instrument (BioRad, Hercules, CA, USA). Levels of gene expression were normalized to GAPDH or U6 small nuclear RNA, an internal control for mRNA and miRNA, respectively. Primers used were FOXM1 (5’-CGTCGGCCACTGATTCTCAAA-3’ and 5’-GGCAGGGGATCTCTTAGGTTC-3’), MALAT1 (5′-AAAGCAAGGTCTCCCCACAAG-3′ and 5′-GGTCTGTGCTAGATCAAAAGGCA-3′), and GAPDH (5’-GCCCAATACGACCAAATCC-3’ and 5’-AGCCACATCGCTCAGACAC-3’). Sequences for U6 RNA (the internal control) and miR-320a were 5’- AAAAGCUGGGUUGAGAGGGCGA-3’ and 5’-GTGCTCGCTTCGGCAGCACATAT-3’, respectively. The expression level of each gene was calculated using the 2−ΔΔCT method.

### Plasmid construction and dual luciferase reporters assay

The binding sites of miR-320a to MALAT1 were first identified by using RNAhybrid 2.234 (https://bibiserv.cebitec.uni-bielefeld.de/rnahybrid/) and the actual binding site sequences of miR-320a to MALAT1 (NR_002819.2) were cloned into pmiRreport (Ambion and Applied Biosystems, Foster City, CA, USA) between *Sac*I and *Mlu*I. pMALAT1(1199) was constructed by cloning MALAT1 (NR_002819.2) nucleotides 360–1620 in pmiRreport between *EcoR*I and *Xho*I. The 3′-untranslational region (3′-UTR) of FOXM1 was amplified from human genomic DNA using PCR and then cloned into a modified pGL3 luciferase vector (Promega, Madison, WI, USA) using the following primers (5′- TGGATCTTGGGTTCTTCACT-3′ and 5′-CATGTCCACCTTCGCTTTTA-3′). The PCR products with the appropriate primers generated inserts with point substitutions in the miRNA complementary sites were also cloned to generate the pC3-FOXM1-mut 3′UTR vector as a mutant control. These constructs were then confirmed by DNA sequencing before use.

For the dual luciferase reporter assay, HEK293T cells were co-transfected with 150 ng of a firefly luciferase reporter plasmid pGL3-MALAT1-Seed1 (Seed1), or pGL3- MALAT1-Seed2 (Seed2) and a renilla luciferase vector (pRL-SV40, Promega) plus small RNAs (miR-101 mimics or negative control RNAs) using Lipofectamine 2000 (Invitrogen). pGL3-FOXM1-UTR-wt and pGL3-FOXM1-UTR-mut (Sangon Biotech, Shanghai, China) were also transfected into HEK293T cell using Lipofectamine 2000. Each experiment was in triplicate and a total of three independent transfection experiments were performed. The firefly luciferase activities derived from pGL3-control-derived plasmids were normalized to renilla luciferase activity from pRL-SV40 using a luciferase assay system (Promega) according to previous studies [37, 38]. The relative luciferase activity was normalized to renilla luciferase activity 48 h after transfection.

### Locked nucleic acid (LNA)-based miRNA microarray assay

Microarray experiments were performed using dual-color hybridizations in locked nucleic acid (LNA) mercury microarrays (Exiqon, Vedbaek, Denmark). To compensate for dye-specific effects, a dye-reversal-color-swap was applied. Briefly, total RNA was isolated using the TRIzol agent (Invitrogen) and miRNeasy mini kit (Qiagen) according to the manufacturers’ instructions and then quantified using a NanoDrop 1000 (Thermo-Fisher Scientific, Waltham, MA, USA) to determine the A260/A280 ratio. RNA samples (2 μg) of each pool of cells were labeled with the miRCURY Hy3/Hy5 Power labeling kit and then hybridized to the miRCURY LNA Array (version 16.0), which contains probes to capture different miRNAs. Data files were further analyzed with the Rosetta Resolver Biosoftware, Build 7.2 (Rosetta Biosoftware, Seattle, WA, USA). A 1.5-fold expression change was used as the cut-off value and the data together with anti-correlation values of the ratio profiles were subjected to the microarray analysis and found to be highly significant (*P*<0.01).

### Cell proliferation assay

Cells were plated at a density of 2000 cells/well in 96-well plates in triplicate and grown overnight. This was then transfected with miR-320a mimics, a miR-320a inhibitor, FOXM1 cDNA, FOXM1 shRNA, negative control RNA (NC), or MALAT1 siRNAs (siM) for 48 h. Cell proliferation rates were monitored for 5 days thereafter with the Cellomics ArrayScan VT1 (Thermo, Waltham, MA, US) according to the manufacturer’s protocols. The presence of a single cell per well was verified by microscopy and monitored for proliferation. Viable clones were expanded and the presence of the fluorescent vector was confirmed using the high-content microscope, which captured images in five fields at each time point. Cell counts were then analyzed for.

### Immunoblotting

Protein was extracted from HUVECs and subjected to immunoblotting analysis of protein levels according to previous studies [29, 30]. The following antibodies were used: a mouse monoclonal anti-β-actin antibody (1: 500, Cat#: A5441, Sigma-Aldrich, St Louis, MO, USA) and a rabbit polyclonal anti-FOXM1 antibody (1:500, Cat#: ab175798, Abcam, Cambridge, MA, USA).

### Statistical analysis

All experiments were performed in triplicate and repeated at least three times. The sample sizes for relevant experiments were determined by statistical power analysis. Data were expressed as the mean ± SEM and analyzed using Student’s *t*-test to compare two groups using the PRISM Software, Version 4 (GraphPad Software, La Jolla, CA, USA). A value of *P*<0.05 was considered to be statistically significant.
